# DDI2 Is a Ubiquitin-Directed Endoprotease Responsible for Cleavage of Transcription Factor NRF1

**DOI:** 10.1016/j.molcel.2020.05.035

**Published:** 2020-07-16

**Authors:** A. Barbara Dirac-Svejstrup, Jane Walker, Peter Faull, Vesela Encheva, Vyacheslav Akimov, Michele Puglia, David Perkins, Sandra Kümper, Suchete S. Hunjan, Blagoy Blagoev, Ambrosius P. Snijders, David J. Powell, Jesper Q. Svejstrup

**Affiliations:** 1 Mechanisms of Transcription Laboratory, The Francis Crick Institute, 1 Midland Road, London NW1 1AT, UK; 2 Protein Analysis and Proteomics Laboratory, The Francis Crick Institute, 1 Midland Road, London NW1 1AT, UK; 3 Department of Biochemistry and Molecular Biology, University of Southern Denmark, 5230 Odense M, Denmark; 4 Crick-GSK Biomedical LinkLabs, GlaxoSmithKline, Gunnels Wood Road, Stevenage SG1 2NY, UK

**Keywords:** Ddi1, DDI2, NRF1, NFE2L1, proteasome, ubiquitin, ubiquitin protease, proteasome inhibition, Bortezomib

## Abstract

The Ddi1/DDI2 proteins are ubiquitin shuttling factors, implicated in a variety of cellular functions. In addition to ubiquitin-binding and ubiquitin-like domains, they contain a conserved region with similarity to retroviral proteases, but whether and how DDI2 functions as a protease has remained unknown. Here, we show that *DDI2* knockout cells are sensitive to proteasome inhibition and accumulate high-molecular weight, ubiquitylated proteins that are poorly degraded by the proteasome. These proteins are targets for the protease activity of purified DDI2. No evidence for DDI2 acting as a de-ubiquitylating enzyme was uncovered, which could suggest that it cleaves the ubiquitylated protein itself. In support of this idea, cleavage of transcription factor NRF1 is known to require DDI2 activity *in vivo*. We show that DDI2 is indeed capable of cleaving NRF1 *in vitro* but only when NRF1 protein is highly poly-ubiquitylated. Together, these data suggest that DDI2 is a ubiquitin-directed endoprotease.

## Highlights

• *DDI2* KO cells accumulate highly ubiquitylated proteins that are slowly degraded
• *DDI2* KO cells are hypersensitive to pharmacological proteasome inhibition
• Purified DDI2 protein can directly cleave the highly ubiquitylated proteins *in vitro*
• DDI2 site-specifically cuts NRF1 *in vitro* but only if NRF1 is poly-ubiquitylated

## Graphical Abstract

**Figure:**
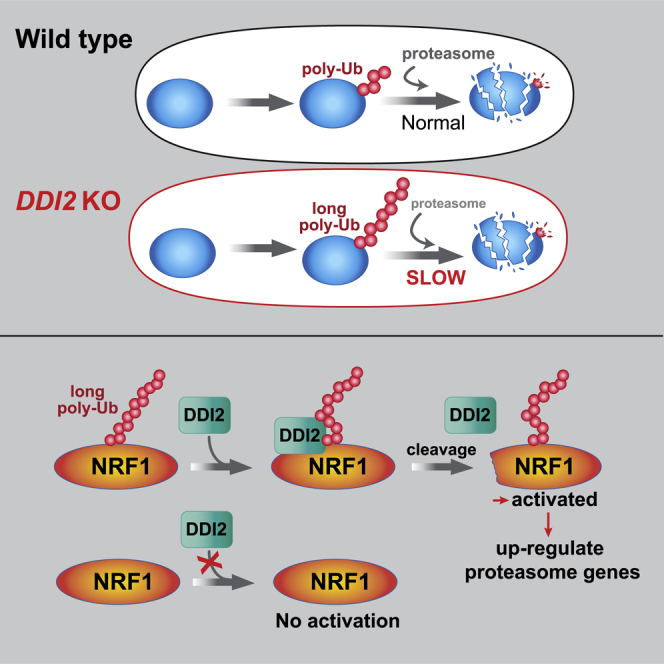

## Teaser

Dirac-Svejstrup et al. show that *DDI2* knockout cells accumulate highly ubiquitylated proteins that are difficult to degrade and that these cells are hypersensitive to pharmacological proteasome inhibition. The DDI2 protein can directly cleave the highly ubiquitylated proteins *in vitro* and does this site specifically in the ubiquitylated transcription factor NRF1.

## Introduction

The ubiquitin-proteasome system (UPS) plays a crucial role in the regulated degradation of proteins (Varshavsky, 2017). As a general rule, UPS targets are poly-ubiquitylated, typically via a lysine 48-linked ubiquitin chain and recognized by ubiquitin receptors on the proteasome that guide them to the proteasome barrel for degradation, during which mono-ubiquitin is released by the action of ubiquitin proteases (also called deubiquitylating enzymes [DUBs]). In some cases, however, so-called ubiquitin shuttling factors are involved in directing proteins to the proteasome as well (Saeki, 2017, Zientara-Rytter and Subramani, 2019). The function of these important UPS factors remains poorly understood.

The main ubiquitin shuttling factors are HRAD23 (Rad23 in budding yeast), Ubiquilin (Dsk2), and DDI2 (Ddi1). In yeast, the ubiquitin shuttling factors all contain a ubiquitin-associated (UBA) domain that binds ubiquitin (Bertolaet et al., 2001b, Nowicka et al., 2015), and a ubiquitin-like (UBL) domain enabling them to bring ubiquitylated substrates to the proteasome (Gabriely et al., 2008, Gomez et al., 2011, Ivantsiv et al., 2006, Kaplun et al., 2005, Zientara-Rytter and Subramani, 2019). Interestingly, the UBA domain is missing in human DDI2, which instead contains a domain with homology to ubiquitin-interacting motifs (UIMs) (Sivá et al., 2016). Moreover, the UBL domains of both yeast Ddi1 and human DDI2 are capable of binding ubiquitin as well (Nowicka et al., 2015, Sivá et al., 2016).

While the functional importance of DDI2 in particular remains relatively poorly understood in metazoans, genetic experiments have indicated an involvement of budding yeast Ddi1 in a variety of cellular functions, including cell-cycle progression, mating type switching, maintenance of genome stability, and protein secretion (see, for example, Gabriely et al., 2008, Ivantsiv et al., 2006, Kaplun et al., 2005, Svoboda et al., 2019, Voloshin et al., 2012, White et al., 2011, Zhu and Xiao, 1998, Zhu and Xiao, 2001). Some yeast *ddi1Δ* phenotypes are exacerbated by concomitant deletion of *RAD23* and *DSK2* (Díaz-Martínez et al., 2006). This and other evidence support the idea that the shuttling factors functionally overlap (Díaz-Martínez et al., 2006, Saeki et al., 2002). Indeed, the yeast Ddi1 and Rad23 proteins are capable of forming a complex (Bertolaet et al., 2001a).

DDI2/Ddi1 is unique among the ubiquitin shuttling factors in also containing a domain with structural similarity to the active site domain of retroviral aspartyl proteases (Sirkis et al., 2006). Remarkably, however, only a single publication has reported DDI2/Ddi1 protease activity *in vitro*, with the purified *Leishmania major* enzyme showing activity at low pH against BSA and some peptide substrates (Perteguer et al., 2013). There has thus generally been a failure to detect DDI2/Ddi1 protease activity *in vitro*, against ubiquitin, ubiquitin chains, or other presumed/likely substrates, although mutation of the active site of DDI2/Ddi1 perturbs its function *in vivo* (see, for example, Gabriely et al., 2008, Koizumi et al., 2016, Lehrbach and Ruvkun, 2016, Svoboda et al., 2019, White et al., 2011).

Recent results in human cells point to a role for DDI proteins in suppressing replication stress through an effect on the stability of replication termination factor 2 (RTF2) (Kottemann et al., 2018), but more is known about the effect of DDI2 on transcription factor NRF1 (NFE2L1) (Motosugi and Murata, 2019). This protein is normally primarily associated with the endoplasmic reticulum (ER), but upon proteasome inhibition, protein processing allows NRF1 to enter the nucleus and upregulate a subset of genes, including those encoding proteasome subunits (Radhakrishnan et al., 2010, Radhakrishnan et al., 2014, Sha and Goldberg, 2014, Vangala et al., 2016). Given the widespread use of proteasome inhibitors in cancer therapy (Roeten et al., 2018), this aspect of DDI2 and NRF1 function has obvious clinical implications. NRF1 is cleaved at a specific sequence motif during activation (Radhakrishnan et al., 2014), and such cleavage requires the DDI2 active site *in vivo* (Koizumi et al., 2016; see also Sha and Goldberg, 2016). The requirement for a DDI family protein in Nrf1 activation is observed in nematodes as well (Lehrbach and Ruvkun, 2016). Intriguingly, however, attempts to reconstitute DDI2-meditated NRF1 cleavage reaction *in vitro* with purified proteins were unsuccessful (Koizumi et al., 2016). So, whether the effect of *DDI2* mutation on NRF1 cleavage is direct and, if so, how DDI2 acts as a protease has remained unclear.

Here, we provide evidence that DDI2 is required for the timely degradation of a subset of ubiquitylated proteins. Indeed, in the absence of *DDI2*, human cells accumulate slowly migrating ubiquitylated proteins, which are substrates for the purified DDI2 protein *in vitro*. Importantly, we show that DDI2 can also cleave its well-known target NRF1, but only when NRF1 is isolated in a highly poly-ubiquitylated form. Together, our data indicate that DDI2 is a ubiquitin-directed endoprotease.

### Results and Discussion

**Figure 1:**
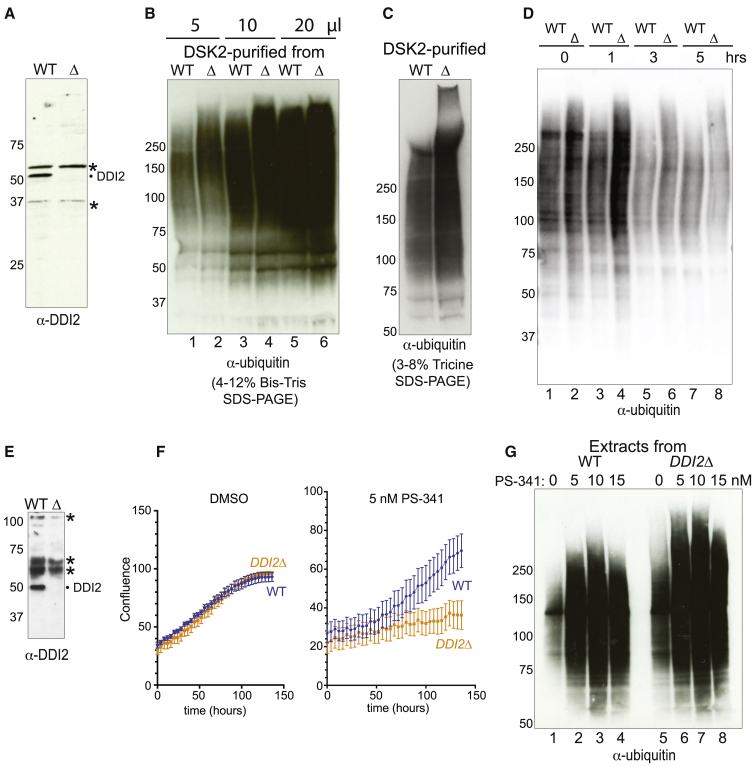
*DDI2* KO Cells Accumulate Slow-Migrating Ubiquitylated Proteins (A) DDI2 western blot analysis of MRC5VA WT and *DDI2* KO (Δ) cells. Asterisks denote non-specific bands. (B) Western blot analysis of ubiquitylated proteins after DSK2 chromatography of extracts from WT and *DDI2* KO (Δ) cells. For ease of comparison between WT and Δ, increasing amounts of protein was loaded. See quantification in Figures S1A–S1C. (C) As in (B), but using another SDS-PAGE gel type, as indicated. (D) Stability of ubiquitylated protein species in WT and *DDI2* KO (Δ) extracts after cycloheximide inhibition of new protein production at time = 0. Gel as in (B). See quantification in Figures S1D and S1E. (E) As in (A), but in U266B cells. (F) Incucyte Live-Cell Analysis of U266B multiple myeloma re-growth after a 16 h treatment with 5 nM PS-341 (Bortezomib) and control (DMSO). Confluence analyzed in Prism. Experiments were performed in triplicate, and numbers represent mean ± SD. See Figure S2 for MRC5VA cells. (G) Western blot analysis of ubiquitylated proteins after treatment of U266B multiple myeloma cells with different concentrations of Bortezomib/PS-341. Cell extracts were analyzed directly, on 4%–15% Criterion TGX gels. See also Figures S1 and S2.

In order to study the function of DDI2, we used CRISPR technology to generate MRC5VA cells in which the *DDI2* gene was knocked out (*DDI2* KO) (Figure 1A). These cells grew normally and showed no outward signs of cellular stress. To study the effect of *DDI2* KO on ubiquitin biology, ubiquitylated proteins were analyzed by western blot analysis, initially after isolation via GST-DSK2 affinity chromatography (Anindya et al., 2007, Tufegdzic Vidakovic et al., 2019). Such proteins migrated as smears upon SDS-PAGE and western blot analysis using anti-ubiquitin antibodies (Figure 1B). Intriguingly, we noticed that ubiquitylated proteins migrated markedly more slowly when isolated from *DDI2* KO cells. Importantly, although comparing the heterogenous smears observed upon anti-ubiquitin western blotting can sometimes be challenging, the differences were highly consistent and independent of the level of protein loading (Figure 1B, compare lanes 2 and 3, for example). Quantification of the differences confirmed the validity of these observations (Figures S1A–S1C). Moreover, we noticed that electrophoresis on BioRad 3%–8% Tricine (or TGX) SDS-PAGE gels gave rise to a more clear-cut readout for these differences, with slowly migrating ubiquitylated proteins from *DDI2* KO cells observed in an area at the top of the gel that was often largely free of signal in wild-type (WT) cells (Figure 1C). These data suggest a role for DDI2 in the processing of a subset of ubiquitylated proteins.

To investigate the possibility that *DDI2* affects the degradation of some ubiquitylated proteins, WT and *DDI2* KO cells were treated with the translation inhibitor cycloheximide, and the fate of ubiquitylated proteins was analyzed at different times over the next several hours. While, as expected, ubiquitylated proteins were generally rapidly degraded in WT cells, the high molecular weight (MW) ubiquitylated species were more persistent in *DDI2* KO cells (Figure 1D; see also the quantification in Figures S1D and S1E), indicating that these proteins are more slowly removed by the UPS.

Recognizing the potential importance of DDI2 in the context of proteasome inhibitor therapy in cancer treatment, we also investigated the effect of *DDI2* KO in the myeloma cell line U266B, which was shown to be sensitive to the first generation of proteasome inhibitors (Hideshima et al., 2001). For this purpose, we generated a U266B *DDI2* KO cell line (Figure 1E). Although this *DDI2* KO showed no significant growth defects under normal conditions, it had significant problems recovering from treatment with proteasome inhibitor PS-341 compared to parental cells (Figure 1F). Similar results were observed in MRC5VA cells, although the return to growth in these cells was slightly faster (Figure S2). Interestingly, compared to WT, the *DDI2* KO cells showed evidence of even higher MW ubiquitylated proteins after proteasome inhibition (Figure 1G, compare lanes 2–4 with 6–8). Moreover, also in multiple myeloma cells, *DDI2* KO has the effect of perturbing the processing of ubiquitylated proteins (Figure 1G, compare lane 1 to lane 5).

Together, these results suggest that human DDI2 assists the UPS and that, in its absence, a cohort of slowly migrating ubiquitylated proteins accumulate. They also suggest an important role for DDI2 in the survival and re-growth of cells after treatment with proteasome inhibitor.

### DDI2 Protease Activity *In Vitro*

To investigate whether the slowly migrating ubiquitylated proteins in *DDI2* KO cells are substrates for DDI2 protease activity, we incubated extracts from these cells with purified DDI proteins from different sources (see Figures S3A and S3B for purified proteins). Yeast Rad23 and Ddi1 were previously shown to form a complex (Bertolaet et al., 2001a), but we failed to find evidence for a stable human DDI2-HRAD23 (RAD23) interaction (Figure S3C). For most *in vitro* protease experiments, we separated the ubiquitylated species by Tricine or TGX SDS-PAGE because this analysis method generally provided a clearer, more “binary” readout of the ubiquitin smears at the top of the gel than other gel types (compare Figures 1B and 1C).

**Figure 2:**
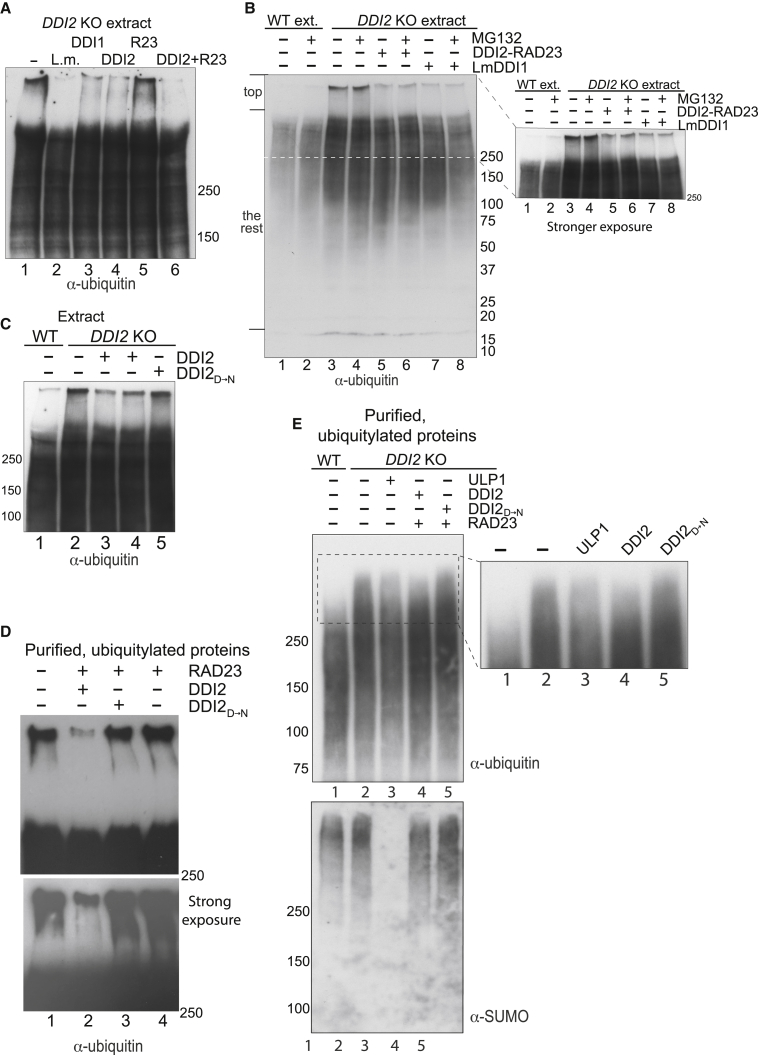
Purified DDI Proteins Digest the Slowly Migrating Ubiquitylated Species from *DDI2* KO Cells (A) Western blot analysis of ubiquitylated proteins after incubation of *DDI2* KO extract with the indicated DDI proteins (human DDI1 and DDI2 from HEK293; HRAD23 (R23) from Mybiosource; *Leishmania major* DDI1 (L.m.) from insect cells; see Figure S3 for purified proteins) (3%–8% Tricine gels). The extent of the effect of RAD23 varies between experiments, but it never has activity on its own. (B) As in (A), but testing the effect of proteasome inhibitor MG132 (4%–15% TGX gel). Right panel is a stronger exposure of the area indicated on the left. Numerous iterations of this experiment have been performed, both in extracts and with purified ubiquitylated proteins, and proteasome inhibition consistently has no effect. See Figure S4 for quantification. (C) As in (A), but testing recombinant DDI2 proteins, all derived from *E. coli*, including DDI2_D→N_ (4%–15% TGX gel). Note that untagged (lane 3) and His-SUMO-tagged (lane 4) WT DDI2 are equally active. (D) As in (C), but using DSK2-purified ubiquitylated substrates. The lower panel is a stronger exposure. Only the top of the gel is shown. See also quantification of an independent experiment in Figures S5A–S5C. (E) As in (D), but including Ulp1 digestion, and probing the same membrane with anti-ubiquitin and anti-SUMO antibodies, as indicated (4%–12% Bis-Tris gel). The panel on the right is an enlargement of the area indicated on the left. See also Figures S3–S5.

Upon incubation of extracts with human or *Leishmania major* DDI proteins, the most clearly visible effect was that the slowly migrating ubiquitylated proteins from *DDI2* KO cells disappeared or greatly diminished (Figure 2A). In support of the functional connection between RAD23 and DDI2 reported by others (Bertolaet et al., 2001a), we found that RAD23 stimulated DDI2-mediated cleavage (Figure 2A, lane 6 versus lane 4; see also Figure 4) but had no effect on its own (lane 5). RAD23 was therefore added to most subsequent experiments. Given that no ATP was added to these reactions, the DDI proteins did not work indirectly by stimulating ATP-dependent degradation by the proteasome in these assays. Indeed, addition of a proteasome inhibitor had little or no effect on the removal of ubiquitylated proteins (Figure 2B, left and right panels). Scanning and quantification of the gel suggested a small, general decrease in ubiquitin signal upon DDI2 addition (Figure S4B), but ubiquitylated proteins of high MW were clearly preferentially affected (Figure S4C). Importantly, highly purified human DDI2 from *E. coli* removed the high MW ubiquitylated proteins as well, and this required the DDI2 active site (Figure 2C, compare WT in lanes 3 and 4 with DDI2_D→N_ mutant in lane 5; DDI2_D→N_ has the catalytic aspartate_252_ in the retropepsin domain mutated to asparagine), further indicating that the effect is direct.

Ubiquitylated proteins are strongly bound to GST-DSK2 resin, allowing their isolation under stringent conditions, including wash in high-salt buffer (Tufegdzic Vidakovic et al., 2019; see also STAR Methods). As in extracts, when such beads containing ubiquitylated proteins were incubated with WT DDI2, a clear reduction in the amount of high MW species was again observed, while DDI2_D→N_ or RAD23 alone had little or no effect (Figure 2D, compare lane 2 with lanes 3 and 4; see also quantification of an independent experiment in Figures S5A–S5C). Other experiments were separated by 4%–12% Bis-Tris PAGE (as in Figure 1B), with similar results (Figure 2E, compare DDI2 treatment in lane 4 with the controls in lanes 2 and 5).

Together, these results indicate that DDI2 is an active protease, capable of digesting the slowly migrating ubiquitylated proteins accumulating in *DDI2 KO* cells.

### Lack of Evidence for a Specific Ubiquitin Chain Type Being Recognized by DDI2

**Figure 3:**
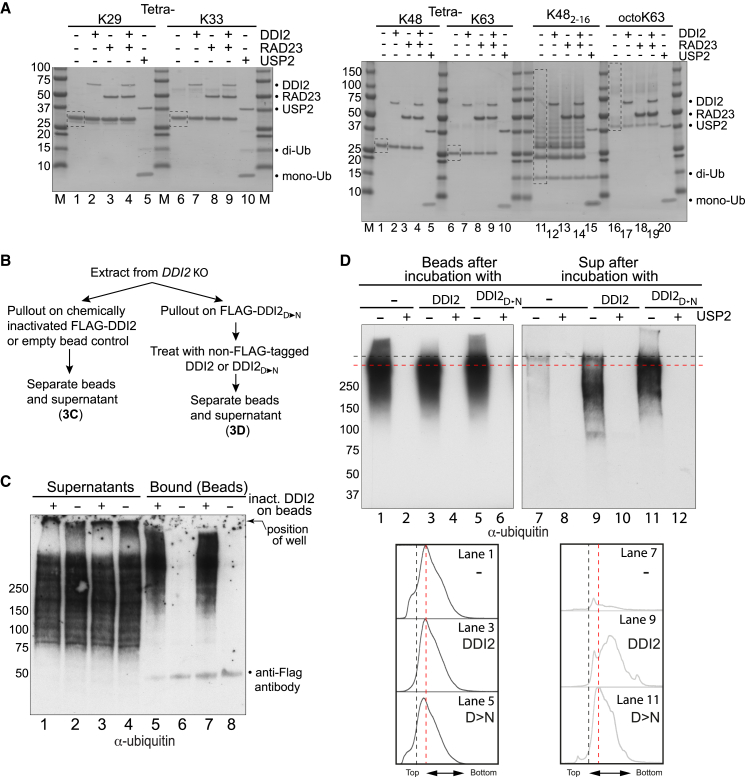
DDI2 Fails to Cleave Purified Ubiquitin Chains but Preferentially Binds and Cleaves Slowly Migrating Ubiquitylated Species (A) Coomassie-stained gels of different commercially available ubiquitin chains before and after incubation with DDI2, RAD23, or the catalytic domain of USP2, as indicated. Untreated substrates are indicated by stippled boxes. Note that USP2 cleaves all these chains to mono- or di-ubiquitin. See also lack of detectable mono-/di-ubiquitin in the blot of Figure S5D. (B) Schematic of experiments in (C) and (D). (C) Western blot analysis of ubiquitylated proteins in bound and unbound (supernatant) fractions after incubation with chemically inactivated DDI2 protein (note that two biological replicates of the same experiment are shown; lanes 1 and 2/5 and 6 and lanes 3 and 4/7 and 8). Note the specific depletion of slowly migrating ubiquitylated proteins in lanes 1 and 3 and the enrichment of the same in lanes 5 and 7. (D) As in (C), but ubiquitylated proteins on FLAG-DDI2_D→N_ beads, incubated with DDI2 or DDI2_D→N_ (non-FLAG tagged to avoid non-specific “displacement” from the beads), as indicated. Note the disappearance of ubiquitylated proteins from beads after incubation with WT DDI2 (above black stippled line, lane 3) and concomitant release of faster-migrating ubiquitylated proteins into the supernatant (below red stippled line, lane 9). See also ImageJ scanning traces below, with position of stippled lines indicated for reference. Please note that the exposure time of the blot on the right is longer than that on the left; only small amounts DDI2-bound material can be eluted. See also Figure S5.

Although DDI2 digestion of ubiquitylated targets did not appear to generate mono-ubiquitin or ubiquitin multimers (Figure S5D; data not shown), we also tested whether our active, purified DDI2 protein is capable of cleaving a range of commercially available ubiquitin chains. DDI2-RAD23 showed no activity against such peptides, including K29- and K33-linked tetra-ubiquitin, as well as K48- and K63-linked tetra- or poly-ubiquitin, even when added in large amounts relative to substrate, while the non-specific catalytic domain of the DUB USP2 (USP2_CD_, here called USP2) efficiently digested the same substrates, mostly to mono-ubiquitin (Figure 3A; data not shown). These results mirror and extend previously reported negative results using the yeast Ddi1 protein (see, for example, Nowicka et al., 2015). Moreover, addition of a large excess of ubiquitin monomer or the different ubiquitin tetramers failed to inhibit the removal of slow migrating ubiquitylated proteins by DDI2 (data not shown).

We also investigated the potential role of SUMOylation. For this purpose, the membrane from the experiment of Figure 2E was also probed with anti-SUMO antibodies (Figure 2E, lower panel). SUMOylation is not a notable characteristic of the slowly migrating ubiquitylated proteins that characterize *DDI2* KO cells: in contrast to ubiquitylation (upper panel), there was no marked difference between WT and *DDI2* KO cells (lower panel, compare lanes 1 and 2). Moreover, while treatment with SUMO protease Ulp1 completely removed the SUMO signal (Figure 2E, lower panel, lane 3), it had no specific effect on the slowly migrating ubiquitylated proteins but instead appeared to generally decrease the signal across the entire cohort of ubiquitylated proteins (upper panel, lane 3). Together, this suggests that DDI2 does not specifically recognize mixed ubiquitin-SUMO chains.

The experiments above focused on the ability of DDI2 activity to remove slowly migrating ubiquitylated proteins from *DDI2* KO extracts. We now performed experiments, outlined in Figure 3B, in which the binding and cleavage substrates of DDI2 were investigated. In order to first look at DDI2 binding without cleavage, we incubated *DDI2* KO cell extract with FLAG-DDI2 in which the active site had been chemically in-activated (DDI2_inact_) and then immobilized this material on M2 beads (Figure 3C, lanes with uneven numbers); empty M2 beads were used as control (even numbers). Elution for western blot analysis was achieved by boiling in loading buffer. Strikingly, the most slowly migrating ubiquitylated proteins were preferentially bound by DDI2_inact_ (Figure 3C, compare bound proteins in lanes 5 and 7 with those in the supernatants of lanes 1–4). Indeed, with a relatively large volume of extract used compared to the volume of DDI2_inact_-affinity beads, the bound fractions were highly concentrated in DDI2-interacting proteins when compared to the extracts in lanes 1–4. A strong signal for high MW proteins was thus found associated with beads (lanes 5 and 7), while these proteins were depleted from the supernatants (compare lanes 1 and 3 with empty beads in lanes 2 and 4). By contrast, faster-migrating species were barely bound at all but were clearly detected in the supernatants. These data support the idea that DDI2 preferentially binds slowly migrating ubiquitylated proteins.

We now investigated whether we could detect cleavage of DDI2-binding proteins. For this purpose, we treated proteins bound to immobilized FLAG-DDI2_D→N_ beads with WT DDI2 in the hope of releasing cleaved proteins into the supernatant, using the inactive DDI2_D→N_ as control (both were non-FLAG tagged) (Figure 3D). Importantly, both western blots and scanning profiles from such experiments consistently showed that the ubiquitylated proteins eluted with active DDI2 migrated significantly faster than those displaced by simple competition with the DDI2_D→N_ control (Figure 3D, compare lane 9 with lane 11 [blot and scan]), indicating that they were indeed cleaved.

Frustratingly, using experiments such as those in Figures 3C and 3D as a starting point, a variety of mass spectrometry approaches failed to uncover convincing and consistent DDI2 binding partners/targets or specific binding determinants of the DDI2-associating, ubiquitylated material (an example is shown in Table S1). However, ubiquitin was consistently detected in such experiments. Moreover, although mass spectrometry did detect the release of ubiquitin, we failed to detect ubiquitin peptides with new amino acid termini that would be indicative of site-specific ubiquitin cleavage after DDI2-mediated digestion.

To investigate the possibility that DDI2 recognizes chains of a specific linkage, or perhaps even branched/forked ubiquitin chains, DDI2-associated material was also subjected to analysis via the UbiSite approach, which allows comprehensive mapping of lysine and N-terminal ubiquitylation sites, including ubiquitin-ubiquitin linkages (Akimov et al., 2018a, Akimov et al., 2018b). Here, ubiquitylated proteins from *DDI2* KO cells purified via immobilized DDI2_D→N_ were compared with the total “ubiquitylome” in WT cells. No significant enrichment in any particular ubiquitin-ubiquitin linkage was observed in the DDI2_D→N_-associated material (Table S2), suggesting that ubiquitin chain composition might not be the major factor in DDI2 substrate recognition.

### DDI2 Cleaves NRF1 in a Ubiquitylation-Dependent Fashion

We now investigated the hypothesis that DDI2 recognizes ubiquitylated proteins and then sometimes cleaves the ubiquitylated protein itself. Upon proteasome inhibition, *DDI2*-dependent processing allows NRF1 to enter the nucleus and upregulate a subset of genes, including proteasome genes (reviewed by Motosugi and Murata, 2019), but whether DDI2 is directly responsible was unclear. Indeed, previous attempts at cleaving NRF1 *in vitro* were unsuccessful (Koizumi et al., 2016).

**Figure 4:**
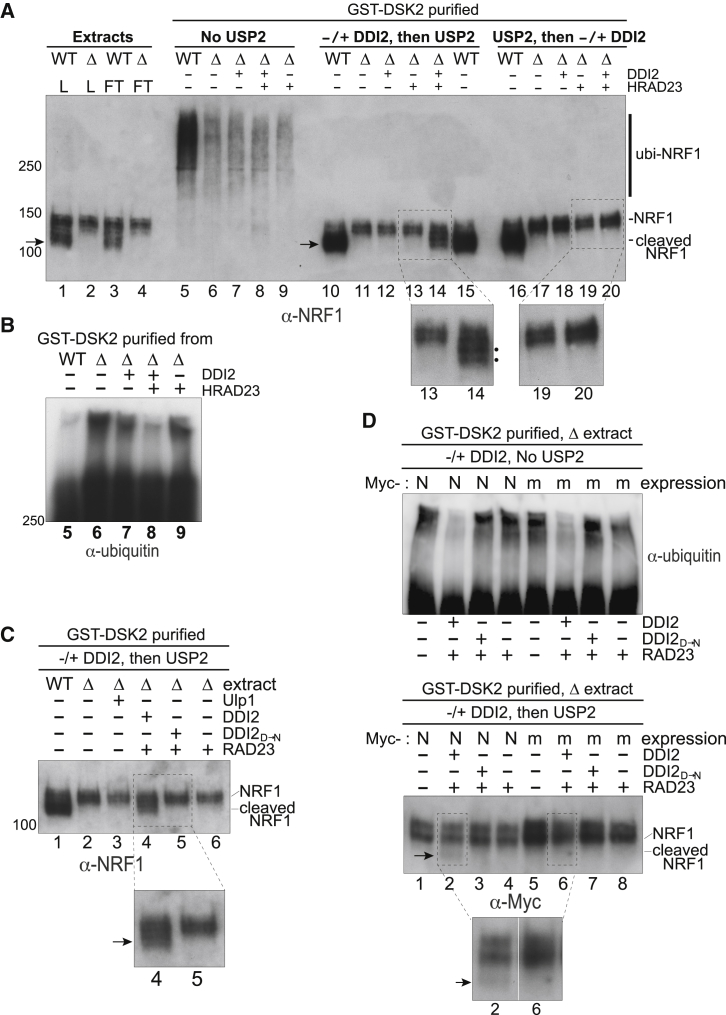
DDI2 Cleaves Ubiquitylated NRF1 Protein *In Vitro* (A) Western blot analysis of NRF1 protein, isolated via GST-DSK2 chromatography, after treatment with DDI2, RAD23, and USP2, as indicated (4%–15% TGX). The mobility of NRF1 and cleaved NRF1 is shown on the right (see also arrow for cleaved NRF1). Zoomed-in images of lanes 13 and 14 and 19 and 20 are shown below. Note that large amounts of extract were used as input for GST-DSK2 purification, meaning that in relative terms, ~20× “extract equivalents” were loaded in lanes 5–20 compared to lanes 1–4. See also Figure S6A. (B) Reprobing of the membrane from (A), with anti-ubiquitin antibodies. For simplicity, only the relevant lanes 5–9 are shown. (C) Experiment as in (A), but testing DDI2_D→N_, as indicated. (D) As in (A) and (B), but testing cleavage of exogenously expressed, Myc-tagged versions of NRF1. N, normal NRF1 sequence. m, mutated sequence. Lower panel, lanes 2 and 6 are shown in the enlargement. The “dot” in lane 6 is not a cleavage band. See also independent experiment in Figure S6B.

Interestingly, when cell extracts were probed using anti-NRF1 antibody, it appears as if NRF1 is not ubiquitylated (Figure 4A, lanes 1 and 2). However, after first enriching for ubiquitylated proteins on DSK2 beads, a smear of very-slow-migrating NRF1 forms becomes evident (lanes 5 and 6). To the best of our knowledge, these forms of NRF1 have not previously been observed, but they must represent poly-ubiquitylated NRF1 as they migrate like the main NRF1 form upon de-ubiquitylation by USP2_CD_ (compare lanes 5 and 6 with 10 and 11). It seems reasonable to speculate that the ubiquitin chains on NRF1 must be very long; indeed, the apparent size difference between the de-ubiquitylated protein and the ubiquitylated species is substantial, with the most slowly migrating forms detected far above the 250 kDa marker (compare lanes 10 and 11 with lanes 5 and 6).

As previously reported by others (Koizumi et al., 2016), WT, but not *DDI2* KO, cells contained the faster-migrating NRF1 band(s) signifying *DDI2*-dependent cleavage *in vivo* (Figure 4A, compare lane 1 [arrow] with lane 2). When fractionated by GST-DSK2 chromatography, these un-ubiquitylated NRF1 species were detected exclusively in the flow-through (FT) fractions (Figure 4A, compare lanes 1 and 2 and 3 and 4 with lanes 5 and 6). Although WT cells generally contained less of the slowly migrating ubiquitylated protein species than *DDI2* KO cells, they contained more ubiquitylated NRF1 protein (compare lanes 5 and 6 between Figures 4A and 4B). The underlying reasons remain to be investigated, but USP2_CD_ digestion of the same samples suggests that a majority of the more abundant, ubiquitylated NRF1 protein in WT extract may actually already be cleaved (Figure 4A, compare lane 10 [arrow] with 11 and with lanes 5 and 6).

No obvious effect was observed after incubation of ubiquitylated NRF1 with DDI2-RAD23 (Figure 4A, lanes 6–9). Importantly, however, NRF1 cleavage products were clearly visible when the reactions were subsequently treated with USP2 to remove all ubiquitylation (Figure 4A, lane 14, compare with lanes 11–13; see also zoomed-in panel). Cleavage required the catalytic activity of DDI2, as DDI2_D→N_ failed to digest NRF1 (Figure 4C, compare lanes 4 and 5). So, the substrate for DDI2 appears to be the ubiquitylated NRF1, with its activity generating endo-proteolytic NRF1 cleavage products reminiscent of those in WT extracts upon ubiquitin removal (Figure 4A, compare lane 14 with lane 15; see also Figure S6A). In agreement with this interpretation, no cleavage was detected if USP2 was allowed to remove ubiquitin chains *prior* to DDI2-RAD23 incubation, showing that DDI2 requires the ubiquitin chain yet cleaves in the body of the NRF1 protein (Figure 4A, compare lanes 17–20 with 11–14). Similar results were obtained with ubiquitylated NRF1 protein isolated via FLAG-DDI2_D→N_ chromatography (Figure S6A), confirming that ubiquitylated NRF1 protein is indeed the substrate for DDI2 activity *in vitro*.

As noted above, the NRF1 cleavage products produced by DDI2-RAD23 were reminiscent of those observed in WT extracts. However, in order to investigate whether NRF1 cleavage requires the same site as previously mapped *in vivo* (Radhakrishnan et al., 2014), transgenes expressing C-terminally Myc-tagged versions of NRF1 containing either the normal (N) or mutant (m) version of the cleavage site (NAWLVH_106_→AAAAAA; “m1” in Radhakrishnan et al., 2014) were transiently expressed in *DDI2* KO cells and treated as above. Significantly, normal NRF1, but not the mutated version, was cleaved by DDI2 *in vitro* (Figure 4D, lower panel, compare lanes 2 and 6 and independent biological replicate in Figure S6B, lanes 2 and 6). This further indicates that the DDI2-mediated NRF1 cleavage reaction *in vitro* faithfully reconstitutes that occurring inside cells. Note that although cleavage of transfected WT NRF1 by DDI2 was very weak in these experiments, digestion of the slowly migrating ubiquitylated species was similar in efficiency to that observed previously (compare Figure 4D, upper panel [ubiquitin blot], lane 2 and lane 6, with Figure 4B, lane 8). The weak cleavage of NRF1 thus likely reflects the fact that the exogenously expressed Myc-tagged version has to compete with endogenous NRF1 in these experiments.

Together, the findings described here can potentially explain the activation of DDI2 and NRF1 upon proteasome inhibition: when proteasome activity is limiting, ubiquitin chains grow very long due to the delay in their degradation. DDI2 recognizes this signal and cuts proteins containing them, such as NRF1, which can then activate proteasome genes. Our results on NRF1 also shed light on the mechanism underlying its activation. Indeed, although it has previously been shown that DDI2 activity is somehow required for cleavage of NRF1 *in vivo*, successful reconstitution of the reaction *in vitro* has not previously been reported.

The slowly migrating ubiquitylated proteins seem to represent a small fraction of total ubiquitylated proteins in *DDI2* KO cells: the fact that these proteins can be detected by western blot analysis might thus be partly explained by the abundance of ubiquitin-antigens in long chains. Indeed, mass spectrometry analysis of the ubiquitylated proteins bound by DDI2 has proven extremely challenging, with even the analysis of their long ubiquitin chains being difficult. The scarcity of ubiquitylated proteins targeted by DDI2 has also hampered our attempts to identify and perform experiments with other targets than NRF1. It is indeed only a very small proportion of the NRF1 pool that carries what must be a very long ubiquitin chain, recognized by DDI2. To the best of our knowledge, this NRF1 form has not previously been visualized, but it is clearly pivotal for DDI2-mediated cleavage and NRF1 function.

We note that our work does not exclude the possibility that DDI2 cuts very long ubiquitin chains as well, for example, in one or a few places rather than at every ubiquitin along the chain. Indeed, it would in many ways make sense in the context of the role of Ddi1/DDI2 as ubiquitin shuttles if infrequent cleavage of long ubiquitin chains facilitated the loading into the proteasome of a large variety of proteins with long ubiquitin chains. Interestingly, while this manuscript was in revision, Rapoport and co-workers reported complementary data that showed that the yeast Ddi1 protein preferentially (or exclusively) recognizes an artificial target protein when it carries a long ubiquitin chain and that it may even weakly cut the chain itself (Yip et al., 2020). Importantly, our results clearly indicate that proteins with very long ubiquitin chains are slowly degraded by the UPS and require DDI2 activity for their timely removal *in vivo*.

In conclusion, the results reported here indicate that DDI2 represents a highly unusual enzyme in the UPS, namely a ubiquitin-directed endoprotease, the only other being the proteasome itself. DDI2 may even be unique in that it appears to also be a ubiquitin-directed, site-specific endoprotease, at least in the case of NRF1 outlined here. Finally, our results, including those on multiple myeloma cells lacking *DDI2*, support the idea that inhibition of DDI2 protease might have clinical benefit in cancer therapy, alone or in conjunction with proteasome inhibition.

## STAR★Methods

### Key Resources Table

**Table:** 

REAGENT or RESOURCE	SOURCE	IDENTIFIER
**Antibodies**		

Anti-Ubiquitin mouse mAb (P4D1)	ENZO Life Sciences	Cat# BML-PW0930; RRID: AB_10998070
Anti-Ubiquitin mouse mAb (P4G7)	ENZO Life Sciences	Cat# ENZ-ABS142; RRID: AB_2331077
Anti-TCF11/NRF1 rabbit mAb (D5B10)	Cell signaling technology Europe BV	Cat# 8052S; RRID: AB_11178947
Anti-mouse secondary (HRP)	Santa Cruz	Cat# sc-516102; RRID: AB_2687626
Anti-rabbit secondary (HRP)	Jackson ImmunoResearch	Cat# 711-035-152; RRID: AB_10015282
Anti-mouse secondary (HRP)	VWR	Cat# NA9310; RRID: AB_772193
Anti-Sumo 2 + Sumo 3 rabbit polyclonal	Abcam	Cat# ab3742; RRID; AB_304041
Anti-Sumo 1 (Y299) rabbit mAb	Abcam	Cat# ab32058; RRID: AB_778173
Anti-DDI2 rabbit polyclonal	Bethyl Laboratories	Cat# A304-629A
Anti-Myc mouse monoclonal (9E10)	Crick laboratories	Evan et al. (1985)

**Bacterial and Virus Strains**		

NEB 5-alpha Competent *E. coli* (High Efficiency)	New England Biolabs	Cat# C2987H
BL21-CodonPlus (DE3)-RIL Competent Cells	Agilent technologies LDA UK LTD	Cat# 230245
XL10-Gold ultracompetent cells	Agilent technologies LDA UK LTD	Cat# 200315
BL21-CodonPlus (DE3)-RIL Competent Cells	Agilent technologies LDA UK LTD	Cat# 230245

**Chemicals, Peptides, and Recombinant Proteins**		

PS-341 (Bortezomib)	Enzo Life Sciences	Cat# BV-1846-1
Recombinant Human USP2 Catalytic Domain, CF	BIO-TECHNE	Cat# E-504-050
Recombinant Human USP2 Catalytic Domain, CF His tagged	CALTAG Medsystems LTD	Cat# AG-40T-0539-C050
Human Rad23 from *E. coli*	MYBIOSOURCE INC	Cat# MBS717584
N-Ethylmaleimide (NEM)	Sigma-Aldrich	Cat# 04260
Ampicillin	Cambridge Bioscience limited	Cat# 2484
MG-132	Sigma-Aldrich	Cat# M7449
L Glutamine	Fisher Scientific UK LTD	Cat# 15410314
Medium, Basal; GIBCO; DMEM (for SILAC)	Fisher Scientific UK LTD	Cat# 12817552
Sodium Pyruvate, GIBCO	Fisher Scientific UK LTD	Cat# 12539059
L-Poline for SILAC	Life Technologies LTD	Cat# 8840
Dimethyl Sulfoxide (DMSO)	Sigma-Aldrich	Cat# D2650
Cycloheximide	2BSCIENTIFIC	Cat# C084-10x1ml
Blasticidin	Thermo-Fisher (GIBCO)	Cat# R21001
Polyornithine	Sigma-Aldrich	Cat# P4957
EPNP (1,2-Epoxy-3-(4-nitrophenoxy)propane: sc. 1,2-Epoxy-3-(4-nitrophenoxy)propane)	Santa Cruz	Cat# sc-258906
Tetra Ubiquitin K48 linked	Boston Biochem	Cat# UC-210B
Tetra Ubiquitin K29 linked	Boston Biochem	Cat# UC-83
Tetra Ubiquitin K63 linked	Boston Biochem	Cat# UC-310B
Tetra Ubiquitin K33 linked	Boston Biochem	Cat# UC-103
Octa Ubiquitin K63 linked	Boston Biochem	Cat# UC-318B
Poly-ubiquitin (Ub2-16) K48 linked	ENZO Life Sciences	Cat# BML-UW0670

**Critical Commercial Assays**		

XT-sample buffer	BioRad Laboratories	Cat# 1610791
Precision PLUS pre-stained markers	BioRad Laboratories	Cat# 1610393
Fetal Bovine Serum Dialysed	Labtech international limited	Cat# FB-1001D
DMEM/F12	Sigma-Aldrich	Cat# D6421
FBS (Australian Origin)	Thermo-Fisher (GIBCO)	Cat# 10099-141
GlutaMAX	Thermo-Fisher (GIBCO)	Cat# 35050-038
Lipofectamine 2000	Thermo-Fisher Scientific	Cat# 1668019
Lipofectamine 3000	Thermo-Fisher Scientific	Cat# L3000015
QIAamp DNA Mini Kit	QIAGEN	Cat# 51306
Q5® High-Fidelity 2X Master Mix	New England BioLabs	Cat# M0492
4-15% TGX gels	BioRad Laboratories	Cat# 5671084
NuPAGE 10% Bis-Tris protein gels	Life Technologies LTD	Cat# NP03031BOX
Criterion XT Tris-Acetate Gel 3-8%	BioRad laboratories	Cat# 3450130
Criterion XT Bis-Tris 4-12%	BioRad Laboratories	Cat# 3450124; Cat# 3450125
Nitrocellulose membrane 0.45 uM	GE Healthcare Life Sciences	Cat# 10600002
Nitrocellulose membrane 0.2 uM	GE Healthcare Life Sciences	Cat# 10600019
Hyperfilm ECL	VWR international	Cat# 29-9068-37
SuperSignal West Pico PLUS ECl reagent	Thermo Scientific	Cat# 34580
DMEM media	Thermo Scientific	Cat# 41966029
RPMI-1640 media	Sigma-Aldrich	Cat# R8758
Radiance ECL HRP substrate	Azure Biosystems	Cat# AC2101
Radiance plus femtogram HRP substrate	Azure Biosystems	Cat# AC2103
Ni-NTA Superflow	QIAGEN LTD	Cat# 30410
Glutathione Sepharose 4B	Sigma-Aldrich	Cat# GE17-0756-01
ANTI-FLAG M2 Affinity Gel	Sigma-Aldrich	Cat# A2220
InstantBlue	Expedeon	Cat# ISB1L
HiTrap Q FF	Fisher scientific UK LTD	Cat# 10607275
HiLoad 16/60 superdex 200	GE health care	Cat# 28-9893-35
XT MOPS running Buffer	BIO-RAD Laboratories	Cat# 1610788
XT Tricine running Buffer	BIO-RAD Laboratories	Cat# 1610790
XT MES running buffer	BIO-RAD Laboratories	Cat# 1610789

**Deposited Data**		

Proteomics (Mass Spec DDI2 interaction)	This study	PRIDE: PXD018215
Proteomics (Mass Spec DDI2 interaction and UbiSite)	This study	PRIDE: PXD019152
Source data (Western blots, etc.)	This study	Mendeley: https://doi.org/10.17632/cz336hbwy8.1

**Experimental Models: Cell Lines**		

Human lung fibroblast cell line MRC5VA	Francis Crick Institute cell depository	N/A
Human lung fibroblast cell line MRC5VA *DDI2* KO	This study	N/A
Human myeloma cell line U266B	Francis Crick Institute cell depository	N/A
Human myeloma cell line U266B *DDI2* KO	This study	N/A

**Oligonucleotides**		

DDI2 gRNA	CGAATAGATTTCAGTAGTAT	N/A
DDI2 Forward PCR Primer	ACTACCATCACCTTCCCCCTA	N/A
DDI2 Reverse PCR Primer	TTGGCAGCAGATAACCTAGGT	N/A
DDI2 Sequencing primer	CTCATTGTTTTTGGCAGCA	N/A

**Recombinant DNA**		

pLenti6/V5-DEST™ Gateway™ Vector	Invitrogen	V49610
pLenti6-DDI2-FLAG	Life Technologies Custom Services	This study
His-Sumo-Rad23A_pET-11a	Genscript	This study
His-Sumo-hDDI2_pET11a	Genscript	This study
His-Sumo-hDDI2_**D→N**__pET11a (D252N)	Genscript	This study
His-Sumo His-Sumo-flag-hDDI2	Genscript	This study
His-Sumo-flag-hDDI2_**D→N**__pET11a (D252N)	Genscript	This study
NRF1-Myc pcDNA3.1(+)-C-Myc	Genscript	This study
NRF1- Myc pcDNA3.1(+)-C-Myc (NAWLVH_106_→AAAAAA)	Genscript	This study
pLenti6/V5-DEST™ Gateway™ Vector	Invitrogen	V49610
pLenti6-DDI2-FLAG	Life Technologies Custom Services	This study

**Software and Algorithms**		

TIDE	Brinkman et al., 2014	https://www.deskgen.com/landing/tide.html#/about
Prism 8 version 8.1.1	GraphPad	https://www.graphpad.com/
ImageJ	NIH	https://imagej.nih.gov/ij/
MaxQuant software version 1.5.3.17	MaxQuant	https://www.maxquant.org/
MaxQuant software version 1.6.0.1	MaxQuant	https://www.maxquant.org/

**Other**		

Neon™ Transfection System	ThermoFisher Scientific	MPK5000
Amersham Imager 600 (AI600)	GE life sciences	N/A
Incucyte	Essenbioscience	https://www.essenbioscience.com/en/products/incucyte/

### Resource Availability

#### Lead Contact

Further information and requests for resources and reagents should be directed to and will be fulfilled by the Lead Contact, Jesper Svejstrup (jesper.svejstrup@crick.ac.uk).

#### Materials Availability

Plasmids will be deposited with and distributed by the non-profit distributor Addgene. There are restrictions to the availability of pLenti6-DDI2-FLAG for human cell expression as it was generated by Life Technologies (now ThermoFisher) through a custom service agreement with GSK, which limits its distribution to third-parties. However, active human DDI2 is now generated in much large quantities using bacterial expression.

#### Data and Code Availability

The mass spectrometry proteomics data have been deposited to the ProteomeXchange Consortium via the PRIDE partner repository with the dataset identifiers PXD018215 and PXD019152.

Original/source data for the figures in the paper is available [Mendeley Data https://doi.org/10.17632/cz336hbwy8.1].

### Experimental Model and Subject Details

#### Cell lines and culture conditions

MRC5VA-derived cells were cultured in high glucose DMEM (Thermo Fisher Scientific, 41966029) supplemented with 10% v/v FBS, 100 U/mL penicillin and 100 μg/mL streptomycin, at 37°C with 5% CO_2_ and routinely passaged 2 times a week. All cell lines were confirmed to be mycoplasma-free. For SILAC Mass Spec experiments, cells were grown in the same manner, but with SILAC media (Basal GIBCO, DMEM Fisher Scientific 12817552), heavy Arginine and/or Lysine with dialysed serum (Labtech international FB-1001D). U266B multiple myeloma cells were cultured in RPMI-1640 (Sigma Aldrich R8758) supplemented with 10% v/v FBS, 100 U/mL penicillin and 100 μg/mL streptomycin, in upright flasks at 37°C with 5% CO_2_ and routinely passaged 2 times a week. 293T cells for overexpression of DDI1 and DDI2 were grown in DMEM/F-12 supplemented with 10% FBS and 2mM GlutaMAX in a 5% CO_2_ incubator at 37°C.

### Method Details

#### Plasmid and lentivirus construction

His-, SUMO- and FLAG-tags were appended to the ORFs for human DDI2 and HRAD23A to generate His-Sumo-Rad23A_pET11a, His-Sumo-DDI2_pET11a, His-Sumo-DDI2_**D→N**__pET11a, His-Sumo-flag-DDI2, His-Sumo-flag-DDI2_**D→N**__pET11a, for protein production and purification. The pET11a cloning sites were NdeI-BamH1, and all plasmids were produced by GenScript. For generation of DDI2-FLAG Lentivirus, a codon-optimized cDNA was first synthesized by GeneArt (Life Technologies), encoding the DDI2 protein with an appended flexible glycine-rich linker and FLAG epitope at the C terminus (GGGGSGGGGSGGGGSDYKDDDDK), and subcloned into pLenti6/V5-DEST Gateway Vector to generate pLenti6-DDI2-FLAG. Generation of a Lentivirus to enable expression of FLAG-tagged DDI2 was carried out using Thermo Fisher Scientific’s ViraPower system through their custom Lentivirus generation service (formerly Life Technologies). pLenti6-DDI2-FLAG was transfected into 293FT cells (Life Technologies) to generate DDI2-FLAG Lentivirus with a titer of 4.9 × 10^6^ (Titer Units/mL as determined by the number of colonies obtained per dilution in a blasticidin resistance assay). For transient transfections of NRF1, the human NRF1 ORF was inserted into pcDNA3.1(+)-C-Myc plasmids, and a mutant version (NAWLVH_106_→AAAAAA) of the mapped cleavage site generated; these were produced by GenScript.

#### Generation of cell lines

*DDI2* knock-out MRC5VA and U266B cells were created using the plasmid pSpCas9(BB)-2A-GFP (PX458 Addgene plasmid 48138). The gRNA sequence is listed in Key Resources. MRC5VA cells were transfected using Lipofectamine 2000 (Thermo Fisher Scientific) according to the manufacturer’s instructions. After 48 h, MRC5VA GFP-positive cells were sorted into 96 well plates by FACS. U266B cells were transfected using the Neon electroporation system (Thermo Fisher Scientific) and GFP-positive cells were collected by FACS as a pool. Cells were allowed to recover and grow for 2 weeks, then sorted into 96-well plates. After growth, cells were lysed in sample buffer and examined by Western Blot using a DDI2 antibody (Abcam). Genomic DNA from potential knock-out cells was isolated using a Qiamp Kit (QIAGEN) and the region around the target site amplified by PCR. The PCR fragment was sequenced and analyzed using the TIDE program (Deskgen) to identify DDI2 KO cells.

#### Growth analysis after Proteasome Inhibitor Treatment

U266B parental and *DDI2* KO cells were seeded in a poly-ornithine coated 96 well plate and treated with 5 nM PS-341 (Bortezomib) in RPMI-1640 medium (Sigma Aldrich R8758) for 16 h. The medium was then carefully removed and cells were washed twice with PBS, then 150 μl RPMI-1640 carefully added to the cells. The plate was incubated in an Incucyte Live Cell Analysis incubator for 6 days and the confluence analyzed in Prism. Likewise, the MRC5VA parental and *DDI2* KO cells were seeded in a 96 well plate at 2 × 10^3^ cells per well and treated with either DMSO or 5nM PS-341 for 16 h, washed twice in PBS and 150ul of DMEM (Life Technologies Ltd) added. The plate was incubated in an Incucyte Live Cell analysis incubator for 6 days and the confluence analyzed in Prism.

#### Myc-NRF1 expression

Plasmids expressing C-terminally Myc-tagged normal or mutant NRF1 were transiently transfected into *DDI2* KO MRC5VA cells, using Invitrogen Lipofectamine 3000. Briefly, 80% confluent cells in 15 cm dishes were incubated with 40 μg WT- or mutant NRF1 pcDNA3.1 (+) -C-Myc plasmid according to the manufacturer’s instruction using 60 μl Lipofectamine 3000 Reagent per ml Opti-MEM Medium. After 24 h, extracts were generated as described below.

#### Protein purification

##### DDI2 expression in human cells, and its purification

293T cells were infected with DDI2-FLAG Lentivirus at a multiplicity of infection (MOI) of 1 in two wells of a 24 well plate. Following overnight incubation, infected cells were selected in media containing 10 μg/mL blasticidin and expanded onto T175 flasks over the course of 10 days. Expression of DDI2 in the resulting non-clonal pool of DDI2-FLAG-expressing cells was confirmed by immunoblot using an anti-DDI2 antibody (Bethyl Laboratories). For DDI2-FLAG purification, 1.75 × 10^8^ cells were seeded into a 10-chamber CellSTACK (Corning), cultured for 4 days following which the cell pellet was harvested and resuspended in ice-cold TBS (150 mM NaCl, 50 mM Tris, pH 7.4). The resuspended cells were lysed by sonication on ice, and insoluble material removed following centrifugation at 100,000 g for 1 h. The FLAG-tagged DDI2 protein was purified on a HiTrap column loaded with anti-FLAG M2 affinity gel using an Äkta purifier (GE Life Sciences). Anti-FLAG bound protein was eluted using 5 column volumes of 150 μg/mL FLAG peptide in TBS. Fractions containing the eluted protein were identified by UV absorbance, collected, run on 4%–20% Tris-Glycine Mini gels (Novex WedgeWell), and Coomassie stained using Instant Blue (Expedeon). Identity of the purified DDI2-FLAG protein was also confirmed by Peptide Mass Fingerprinting.

##### *E.coli* expression and protein purification

Recombinant DDI2, DDI2_**D→N**_ and HRAD23 proteins with various tags were generated from plasmids transformed into BL21 *E.coli* (Agilent Technologies 130245), expressed by IPTG induction at 1 mM for 4 h at 30°C. All proteins contained a His-Sumo-tag; but some also His-Sumo-FLAG, allowing non-tagged and FLAG-tagged versions to be generated by cleavage with the SUMO protease, Ulp1. Bacteria were harvested by centrifugation, washed in PBS and resuspended in PBS with 10 mM Imidazole, Protease inhibitors and 0.1 mg/mL lysozyme followed by sonication (2 min on, 15 s on 20 s off, 20% amplitude Branson sonicator). Cleared lysates were added to Ni-agarose and incubated at 4°C overnight; the beads were washed in 50 mM Tris, pH 8, 10 mM Imidazole, 500 mM NaCl, 10% Glycerol (high salt buffer), then in the same buffer at 150 mM NaCl (low salt buffer), and the His-tagged protein was eluted with low salt buffer containing 300 mM Imidazole. The nickel-purified protein was diluted 1:1 with water and further purified on HiTrap Q FF (Fisher Scientific 10207275), using a 10-column volume gradient from 150 mM (Buffer A) to 1 M NaCl (Buffer B) in 25 mM Tris pH 8, 1% Glycerol, 0.1 mM DTT and Protease inhibitors (2.2 mM PMSF, 2 mM Benzamidine HCL, 2 μM Leupeptin, 1 μg/mL Pep statin A). For removal of the His-SUMO tag, the purified proteins were treated with Ulp1 (a kind gift from Peter Cherepanov) in buffer A for 30 min at 30°C. Proteins were further purified by gel filtration on HiLoad 16/60 Superdex 200 (GE health care 28-9893-35) in buffer A. No marked difference in the activity of His-SUMO-DDI2, His-SUMO-FLAG-DDI2, FLAG-DDI2 (His-SUMO tag removed) and DDI2 (no tag) was detected (Figure 2C, and data not shown).

#### Human cell extract preparation

For preparing whole cell extracts, WT or *DDI2* KO MRC5VA grown on 15 cm dishes to 80%–90% confluency, were rinsed twice with ice-cold PBS containing Protease Inhibitors (2.2 mM PMSF, 2 mM Benzamidine HCL, 2 μM Leupeptin, 1 μg/mL Pep statin A) and 2 mM N-elthylmaleimide (NEM). Cells were then lysed on the plate in 1.5 mL TENT lysis buffer (50 mM Tris-HCl pH 7.4, 150 mM NaCl, 2 mM EDTA, 1% (v/v) Triton X-100), containing 2 mM NEM and Protease inhibitors, for 5 min at room temperature (RT). The lysates were collected in 15 mL falcon tubes and kept on ice for 20 min followed by a brief sonication; 10 s at 20% amplitude in a Branson sonicator. Debris was removed by centrifugation 5 min at 20.000 RCF in an Eppendorf microfuge. The extracts were either used directly or snap-frozen in liquid nitrogen. Extracts were used for affinity purifications or directly in biochemical assays (see below).

#### Western blot analysis

Approximately, 50 μg protein/lane was separated on 4%–12% Bis-Tris Criterion XT (BioRad 3450124/5), 3%–8% Tris-Acetate Criterion XT (BioRad, 3450130), or 4%–15% TGX gels (BioRad, 56711084/5), and transferred to nitrocellulose membranes (GE Healthcare Life Sciences, 10600002, 10600019). Membranes were stained with Ponceau S, scanned and the membranes blocked in 6% (w/v) skimmed milk in PBS for 1 h at room temperature and incubated with primary antibody (in 6% (w/v) skimmed milk in PBS-T or 5% BSA) for 1 h at RT or overnight at 4°C. Primary antibodies are listed in Key Recourses Table. For anti-ubiquitin blots, an equal mixture of P4D1 and P4G7 were used. For SUMO, an equal mixture of Anti-Sumo2 + Sumo3 and Anti-Sumo1 were used. All antibodies were used at 1:1000 dilution, except anti-DDI2 (1:5000). Membranes were washed several times in PBS-T (PBS, 0.1% Tween20 (w/v)), incubated with HRP-conjugated secondary antibody (key resources) in 6% (w/v) skimmed milk in PBS-T for 1 h at RT, washed again, and visualized using SuperSignal West Pico PLUS or Radiance ECL reagents (Azure Biosystems, AC2101,3). Blots were visualized on Amersham hyperfilm ECL or by AI600 Amersham Imager chemiluminescence. Quantification was performed using ImageJ.

#### GST-DSK2 and FLAG-DDI2_D→N_ affinity chromatography

Human cell extracts were generated as described above. GST-DSK2 beads were generated as described (Tufegdzic Vidakovic et al., 2019). Typically, 1 mL cell extract at 1 mg/mL was incubated with 50 μl DSK2 beads rotating overnight at 4°C. Unbound material was saved and the beads were washed three times in 1 mL TENT buffer. Where beads were to be treated with DDI2, they were also washed with high salt TENT buffer (500 mM NaCl) and then washed back into in TENT buffer. Beads were then used for biochemical reactions.

Two methods of Flag-DDI2_inactive_ affinity binding were carried out, either using chemically inactivated DDI2 (see below) or DDI2_D→N_; in both cases, the extracts were pre-incubated for 2 h at 4°C with M2 anti-flag agarose (Sigma-Aldrich 30410) to remove non-specific M2-resin binding proteins. The pre-cleared extracts were then incubated with Flag-DDI2_inactive_ protein, and fresh M2-anti-flag agarose. For some biochemical experiments, an excess of Flag-DDI2_D→N_ was first bound to the beads, the beads washed several times; and then incubated with the cell extracts in TENT buffer. For their use in DDI2 cleavage assays, these beads were treated as described above for DSK-beads, except extract binding to the Flag-DDI2_D→N_ affinity beads was for 4°C for 4 h, and the resin washed in TENT buffer, then with TENT buffer with 500mM NaCl, then twice with TENT buffer before being used for cleavage assays.

#### Chemical inactivation of flag-hDDI2

The DDI2 protease domain comprises a highly conserved retropepsin domain, otherwise best exemplified by HIV protease, which is structurally highly similar to DDI2 (Sivá et al., 2016). 1,2-Epoxy-3-(4-nitrophenoxy) propane (EPNP) is a widely used, mechanistic inhibitor of retropepsins and other aspartic proteases, and potently inactivates HIV protease (Meek et al., 1989). To similarly inactivate DDI2, 100 μL of 1 mg/mL DDI2 was incubated with 10 mM EPNP for 4 h at room temperature, subsequently diluted into PBS (10 ml), and re-concentrated using VIVAspin 500 column (GE Healthcare) with a 10 kDa molecular weight cut-off. A control active DDI2-FLAG preparation without EPNP treatment was processed alongside. Final protein concentrations were determined by bicinchoninic acid (BCA) assay. The chemically inactivated protein was used for the experiments in Figure 3C.

#### DDI2 protease assays

Human cell extracts, ubiquitin peptides, and ubiquitylated proteins isolated by affinity chromatography (see above), were treated with enzymes as described below.

For Ddi1/DDI2 treatment of extracts, approximately 100 μg of human cell extract in TENT buffer was incubated directly with 1-3 μg DDI2 protein in the presence or absence of the same amount of HRAD23 protein at 26, 30, or 37°C; for 30-60 min. Reactions were stopped by adding SDS-PAGE loading buffer. The results were analyzed by SDS-PAGE followed by western blotting.

Ubiquitin tetramers K29, K33, K48, K63 (Boston Biochem UC-83, UC-103, UC-210B, UC-318B) (2ug), K48 polymers Ub2-16 (8 μg), and K63 octamers (4 μg) were treated with 2-4 μg DDI2 and/or 2-4 μg HRAD23 or Usp2_**CD**_ (Bio-Techne E-504-050) for 45 min at 37°C in cleavage reaction buffer (25 mM Tris-HCl pH 7.4, 150 mM NaCl, 1 mM EDTA, 0.25% (v/v) Triton X-100, 5 mM DTT). Reactions were stopped by adding SDS-PAGE loading buffer (LB: XT sample buffer BioRad (1610791) with 0.1M DTT and 10% V/V BME), and the samples analyzed by SDS-PAGE followed by Coomassie (instant blue) staining.

For affinity-purified substrates on beads, incubations were carried out in shaking thermomixers. Beads were incubated in the presence or absence of DDI2 and HRAD23. Beads were also treated with USP2_CD_ (USP2) pre- or post-cleavage. Substrates were also treated with Ulp1 protein Figures 2E and 4C. For cleavage reactions on DSK2 beads, ubiquitylated proteins were typically 20-fold concentrated onto the beads, which were stringently washed, as described above. 20 μl of beads were treated with 3 μg of DDI2 and/or the same amount of HRAD23 in bead cleavage buffer (25 mM Tris-HCl pH 7.4, 120 mM NaCl, 0.5 mM EDTA, 0.25% (v/v) Triton X-100, 5 mM DTT) at 30°C for 1 h. USP2 digests were for 30 min at 37°C. Reactions were stopped adding SDS-PAGE loading buffer, and the results analyzed by SDS-PAGE followed by western blot.

#### Mass spectrometry analysis

For mass-spectrometry of ubiquitylated proteins associated with DDI2, proteins bound to FLAG-DDI2_inact_ were subjected to mass spectrometry (data from proteins in Figure 3C, lanes 5-8 shown in Table S1). Briefly, *DDI2* KO cells were labeled either heavy or light using stable isotope labeling with amino acids in cell culture (SILAC; Ong et al., 2002). Cell extracts were then subjected to affinity chromatography using immobilized, catalytically inactive DDI2 protein. Bound proteins were eluted with SDS-loading buffer or by proteolysis with DDI2. Forward and reverse SILAC mixtures were created by mixing the eluates and subjecting them to SDS-PAGE. Next, the gel lanes were cut from the top to the bottom into multiple different molecular weight regions and prepared for mass spectrometry analysis using an in-gel trypsin digestion protocol. Mass spectrometry data were acquired using an Ultimate3000 HPLC connected to Lumos-Tribrid Orbitrap operated in Data Dependent Acquisition mode. Heavy to light protein ratios for each gel slice were determined using Maxquant software version 1.6.0.1 whereby a shift in heavy/light ratio indicates DDI2 dependent proteolysis (Table S1; see also source data at PRIDE PXD018215). In a second round of searches using the same raw data files, the enzyme specificity setting was changed from tryptic to semi-tryptic to search for novel proteolytic cleavage sites.

#### Analysis of ubiquitylation (UbiSite)

Ubiquitylated proteins purified from *DDI2* KO cells and the total ubiquitylome in WT cells were compared. Ubiquitylated proteins from FLAG-DDI2_D→N_ beads or GST-DSK2 resin were eluted with 8M Guanidine-HCl and subjected to reduction, alkylation and digestion with Lysyl Endopeptidase (LysC) (Wako) followed by enrichment of ubiquitinated peptides using the UbiSite approach exactly as described (Akimov et al., 2018a). 90% of the samples were used for the High pH reversed-phase fractionation (HpH) to reduce samples’ complexity by a stepwise elution of peptides with 1.75, 3.5, 7, 8, 9, 10.5, 14 and 50% of acetonitrile. The remaining 10% of each sample was subjected to parallel reaction monitoring (PRM) for targeted quantitative analyses.

Tryptic peptides from each HpH fraction were injected into a 20-cm fused silica column with an inner diameter of 75 μm packed in house with C18 resin (1.9-μm beads, Reprosil, Dr. Maisch) using an EASY-nLC 1000 chromatography system (Thermo Fisher Scientific) connected online to a Q Exactive HF-X mass spectrometer (Thermo Fisher Scientific) assembled with a nano-electrospray ion source (Thermo Fisher Scientific). Peptides were loaded in solvent A (0.5% acetic acid) and eluted with a gradient of solvent B (80% ACN, 0.5% acetic acid) from 7% to 12% solvent B over 8 min, from 12% to 33% over 90 min, followed by increasing solvent B to 45% for 10 min and finished by a run with 98% for 6 min at 250 nL/min. The Q Exactive HF-X mass spectrometer was operated in positive polarity mode with spray voltage set to 2.3 kV and heated capillary temperature at 275 °C. MS data were acquired using a data-dependent method switching between full scan events and the top 12 MS/MS scans. An automatic gain control (AGC) target value was set to to 3 × 10^6^ and resolution was set to 60,000 for full MS scan events with a scan range of 300–1,700 m/z and a maximum ion injection time (IT) of 15 ms. Precursors were fragmented by higher-energy collisional dissociation (HCD) with a normalized collisional energy (NCE) of 28. MS/MS scans were acquired with a resolution of 30,000, maximum IT of 45ms, 1.2 m/z isolation window. Repeat sequencing of peptides was minimized by setting the dynamic exclusion window to 60 s.

Raw MS data were searched using MaxQuant software v 1.5.3.17 with Andromeda search engine and FASTA file from UniProt released July 2015 (42 127 reviewed sequences), supplemented with commonly observed contaminants. The following search parameters were used: the enzyme used for digestion was specified as trypsin with up to three missed cleavages. Fixed modification was cysteine carbamidomethylation and variable modifications were oxidation of methionine and di-Glycine on lysine residues, excluding lysines on the C-terminal end of peptides. Spectra were searched with a mass accuracy of 4.5 ppm for precursors and 20 ppm for fragment ions. False discovery rate (FDR) was set to 0.01, both at protein and peptide levels, using a reverse database as a decoy.

PRM analyses and quantitation of ubiquitin chains were performed as described previously (Akimov et al., 2018b) with minor modifications, including the specific monitoring of the three peptide sequences TLTGK(gg)TITLEVEPSDTIENVK(gg)AK, TITLEVEPSDTIENVK(gg)AK(gg)IQDK and AK(gg)IQDK(gg)EGIPPDQQR, corresponding to the branched ubiquitin chains at positions K11+K27, K27+K29 and K29+K33, respectively (Table S2; see also source data at PRIDE: PXD019152).

### Quantification and Statistical Analysis

Statistical details of experiments can be found in the relevant figure legends.

## Supplemental Information

Document S1. Figures S1–S6

Table S1. Mass Spectrometry of Ubiquitylated Proteins Associated with DDI2, Related to Figure 3

Table S2. Analysis of Ubiquitylation by UbiSite, Related to Figure 3

Document S2. Article plus Supplemental Information
