# Supplementary material for: DDI2 Is a Ubiquitin-Directed Endoprotease Responsible for Cleavage of Transcription Factor NRF1
Source: Mol Cell. 2020 Jul 16;79(2):332–341.e7. doi: 10.1016/j.molcel.2020.05.035 (PMC7369636; doi:10.1016/j.molcel.2020.05.035)
Supplement: Document S1. Figures S1–S6 [file mmc1.pdf]

Molecular Cell, Volume *79*

Supplemental Information

DDI2 Is a Ubiquitin-Directed

Endoprotease Responsible for Cleavage

of Transcription Factor NRF1

A. Barbara Dirac-Svejstrup, Jane Walker, Peter Faull, Vesela Encheva, Vyacheslav Akimov, Michele Puglia, David Perkins, Sandra Kümper, Suchete S. Hunjan, Blagoy Blagoev, Ambrosius P. Snijders, David J. Powell, and Jesper Q. Svejstrup

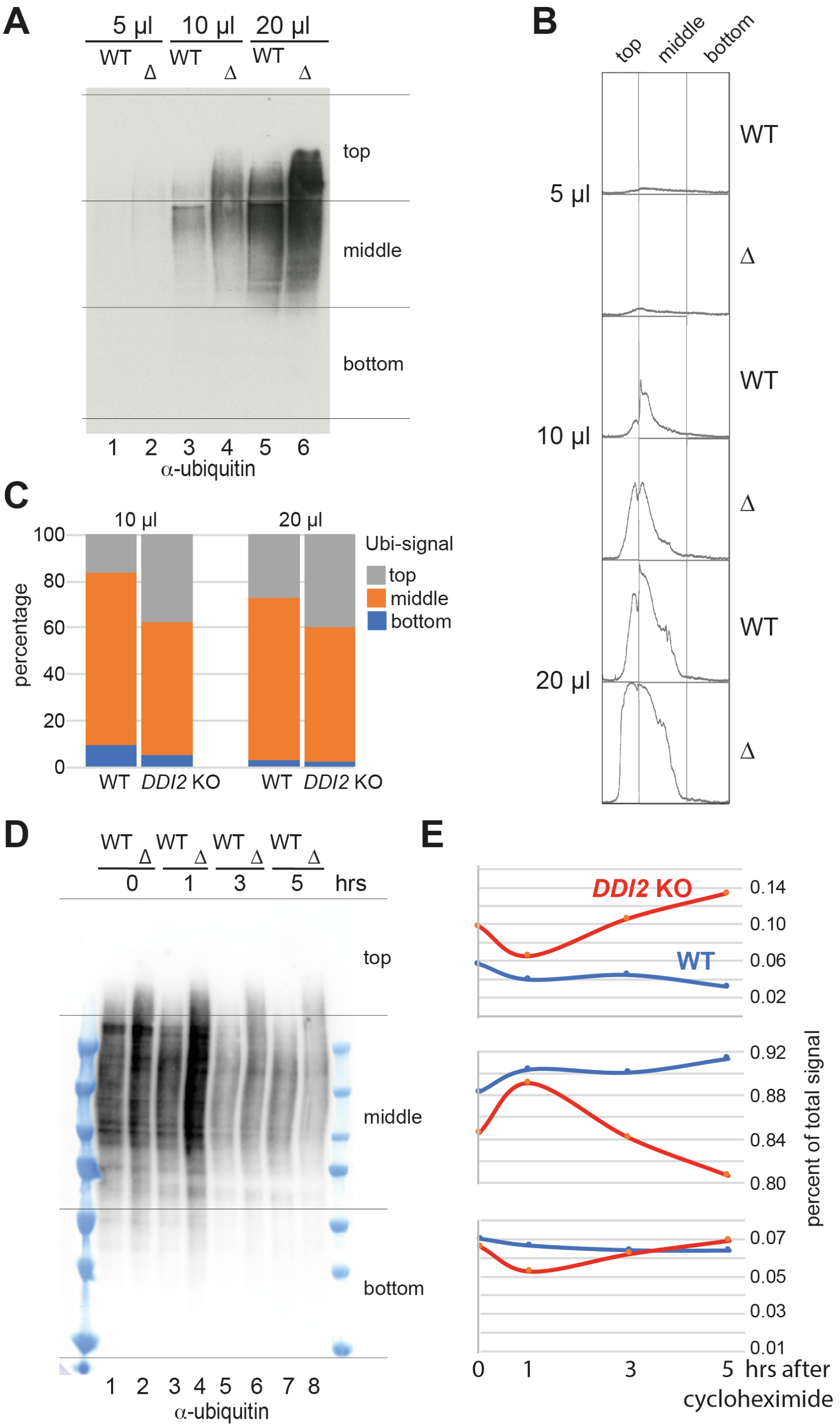

Supplemental Figure S1, related to Figure 1. Quantification of ubiquitylated proteins in *DDI2* KO cells. A. Weak exposure of the gel from Figure 1B, used for scanning and Image J quantification. Δ, *DDI2* KO. **B.** Image J quantification of individual lanes from A. The areas defined as 'top', 'middle' and 'bottom' are also indicated to the right of the gel in A. **C.** Relative proportions of the total lane signal found in the sections of the gel in A., with 10 and 20 µl extract loaded. Note the much higher proportion of material in the upper area (grey) of the gel in the *DDI2* KO. **D.** The gel from Figure 1D, with the areas defined as 'top', 'middle' and 'bottom' indicated. **E.** Graph representing the relative amount of signal in the areas defined in D, over time. Notice that ubiquitylated proteins 'decay' with a similar rate (the curves are flat) in the different regions of the gel in WT cells, while the relative proportion of slowly migrating, ubiquitylated proteins (top) increases, and those in the middle of the gel decreases, in *DDI2* KO cells. Similar trends were observed in other, independent experiments, but differences in gel running, etc., means that it is not meaningful to provide the quantifications with error bars. Those shown are representative of at least two independent experiments. All quantifications performed in Image J.

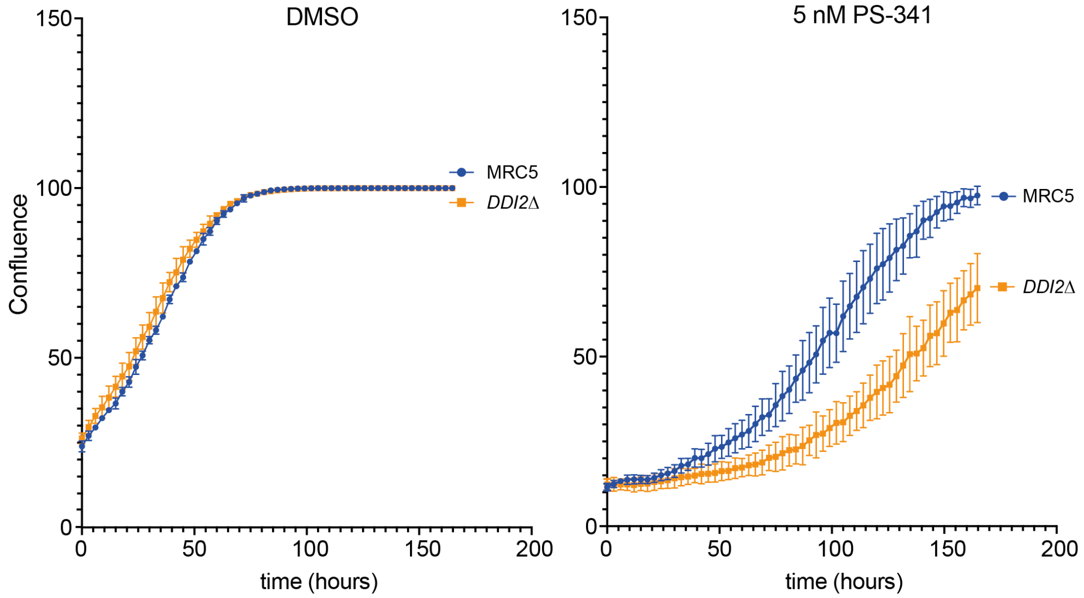

Supplemental Figure S2, related to Figure 1. Re-growth of MRC5VA cells after proteasome inhibtion. Incucyte Live Cell Analysis of parental (MRC5VA) and *DDI2* KO regrowth after a 16 hr treatment with 5 nM PS-341 (Bortezomib), with DMSO as control. Confluence analysed in Prism. Experiments were performed in triplicate, and numbers represent mean+/-SD. See Figure 1 for U266B cells.

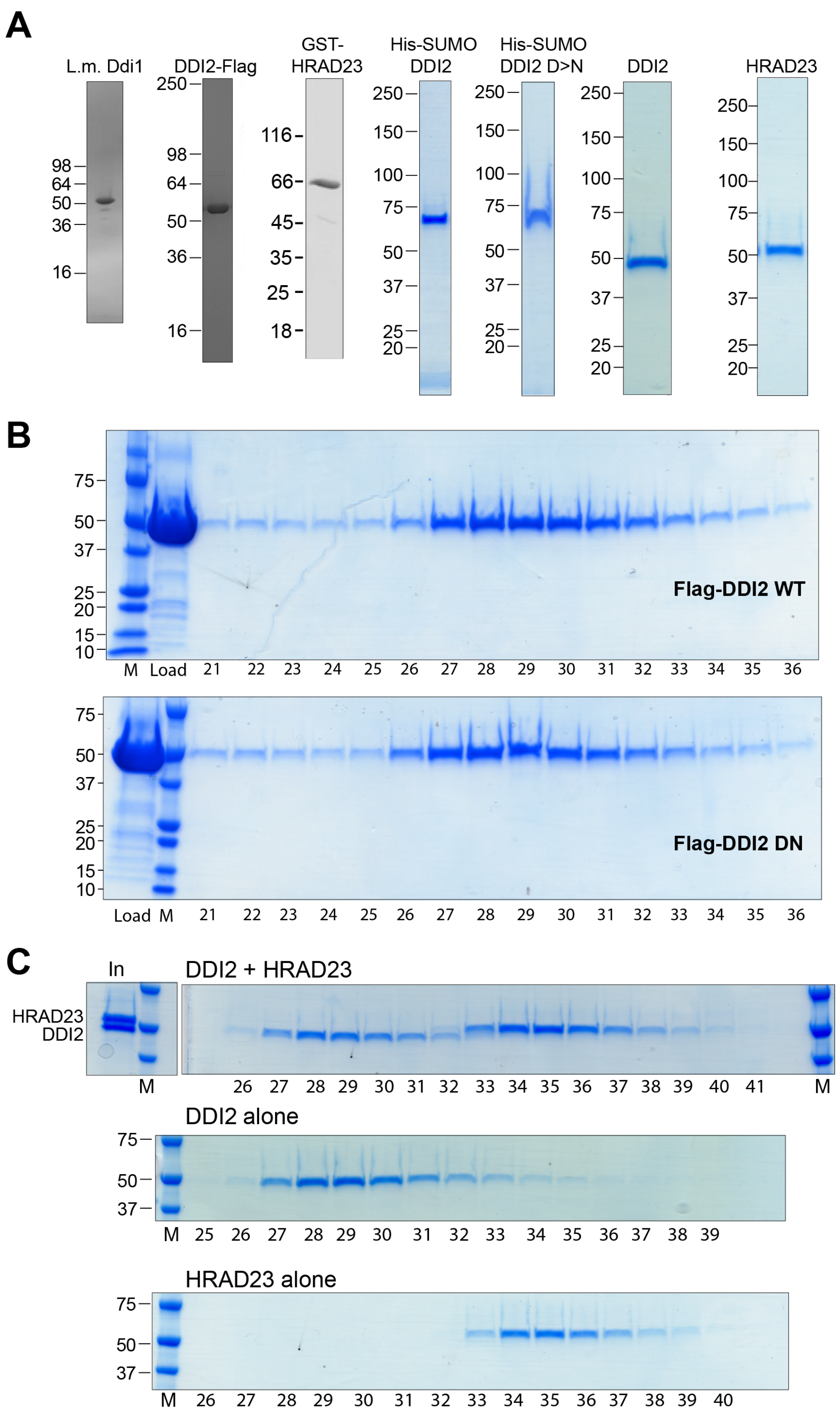

Supplemental Figure S3, related to Figures 2-4. Purified proteins and gel-filtration analysis of DDI2-HRAD23 interaction. A. Purified proteins used in this study: *Leishmania major* His-Ddi1 (recombinant, from insect cells); DDI2-Flag (over-expressed in, and purified from, human HEK293 cells); GST-HHR23A (HRAD23) (commercial source: MyBioSource MBS717584); His-SUMO DDI2 and $DDI2_{D\rightarrow N}$ (recombinant, derived from *E.coli*); untagged DDI2 (recombinant, derived from *E.coli*); HR23A (recombinant, derived from *E.coli*). See StarMethods for details on preparation. **B.** Gel-filtration (Superdex-200) profiles of FLAG-tagged, recombinant (*E.coli*) DDI2 and $DDI2_{D\rightarrow N}$. **C.** Gel-filtration (Superdex-200) profiles of un-tagged, recombinant (*E.coli*) DDI2 and RAD23, alone or together, as indicated.

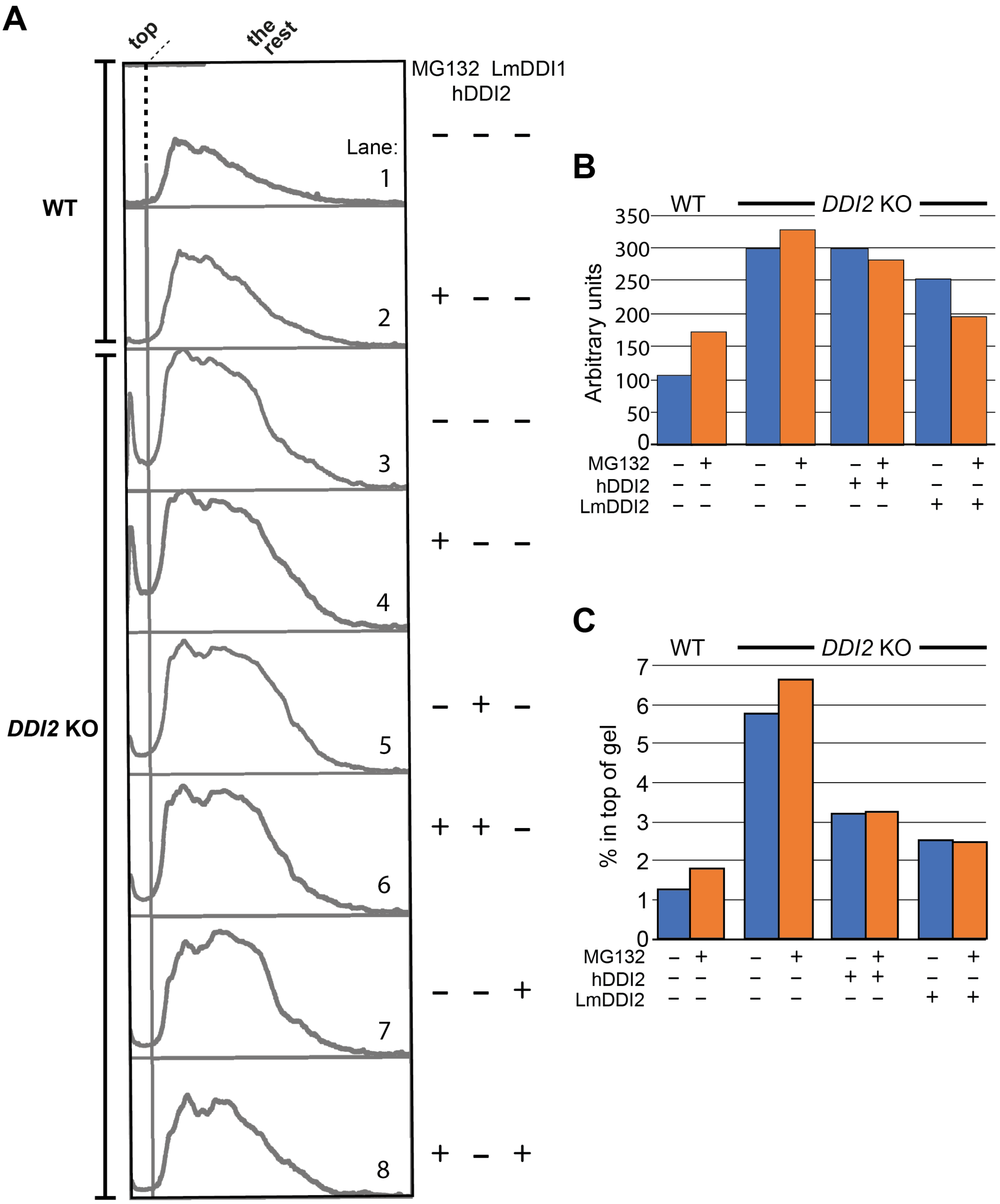

Supplemental Figure S4, related to Figure 2. Quantification of ubiquitylated proteins after DDI treatment in the presence or absence of MG132. A. Image J quantification of ubiquitin signal in the individual lanes from Figure 2B. The areas defined as 'top', and 'the rest' are indicated, also on the gel (see 2B). **B.** The total signal (in arbitrary units) in the different lanes of the gel. **C.** The signal (in percent of total signal in that lane) in the different lanes of the gel. Note that the decrease after treatment with DDI protein is much clearer at the top of the gel compared to the lane overall (B.). Similar trends were observed in other, independent experiments, but differences in gel running, etc., means that it is not meaningful to provide quantifications with error bars. Those shown are representative of at least two independent experiments. All quantifications performed in Image J.

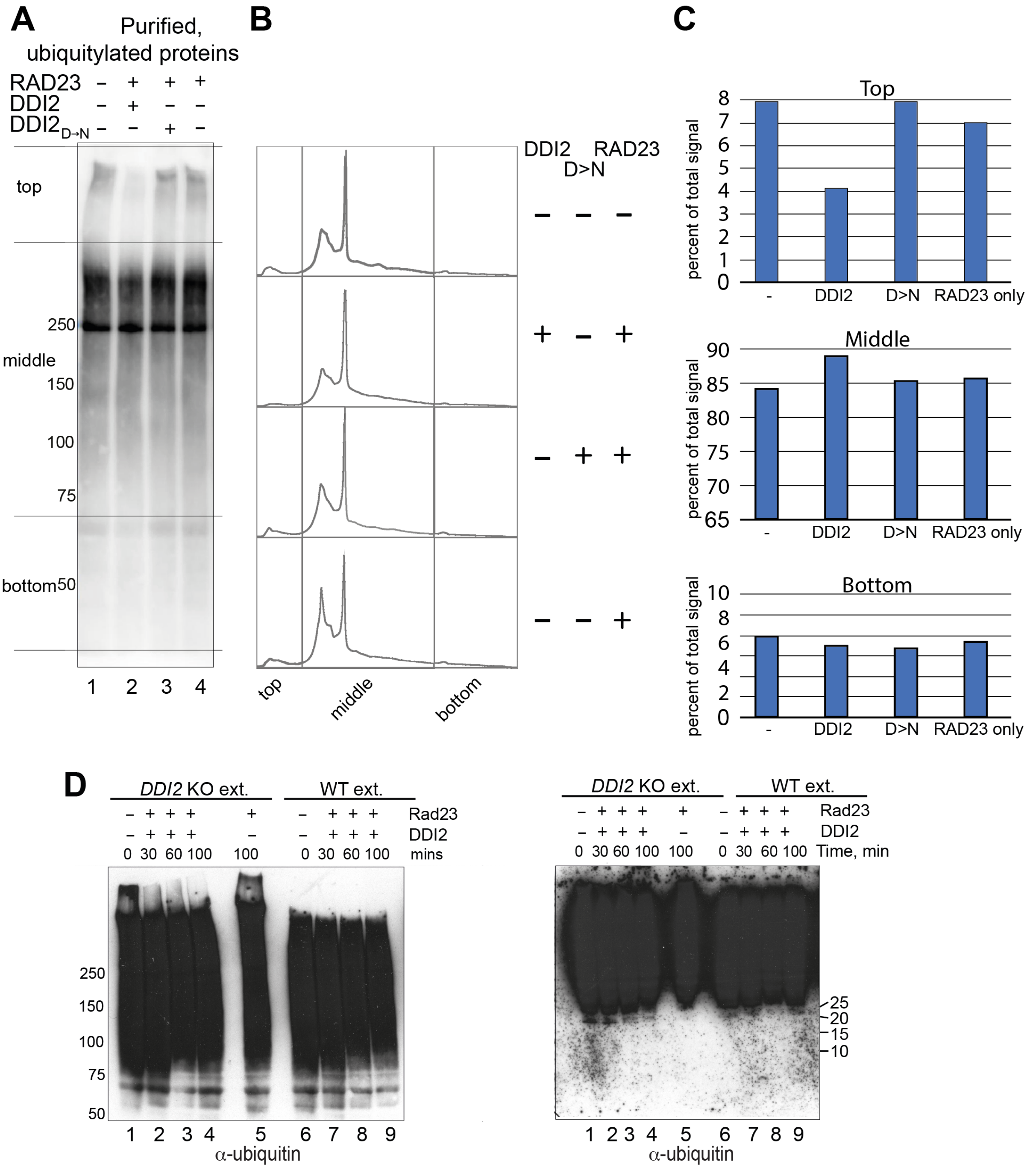

Supplemental Figure S5, related to Figure 2 and 3. Ubiquitylated proteins after DDI2 treatment. A. Gel from independent experiment to that shown in Figure 2D used for quantification (in B. and C.). **B.** Image J quantification of ubiquitin signal in the individual lanes from A. The areas defined as 'top', 'middle' and 'bottom' are indicated, also on the gel in A. **C.** The signal (in percent of total signal in that lane) in the different areas of the gel. Note the clear decrease in signal at the top of the gel after treatment with WT DDI protein only, and the concomitant increase in signal in the middle. Very similar trends were observed in other, independent experiments, but differences in gel running, etc., means that it is not meaningful to provide quantifications with error bars. Those shown are representative of at least two independent experiments. **D.** Time-course of DDI2 activity. Same samples loaded in the gels shown on the left (4-15% BioRad TGX gel) and right (BioRad Bis-Tris 12% in MES buffer; a strong exposure is shown). Similar trends were observed in other, independent experiments, but differences in gel running, etc., means that it is not meaningful to provide quantifications with error bars. Those shown are representative of at least two independent experiments. All quantifications performed in Image J.

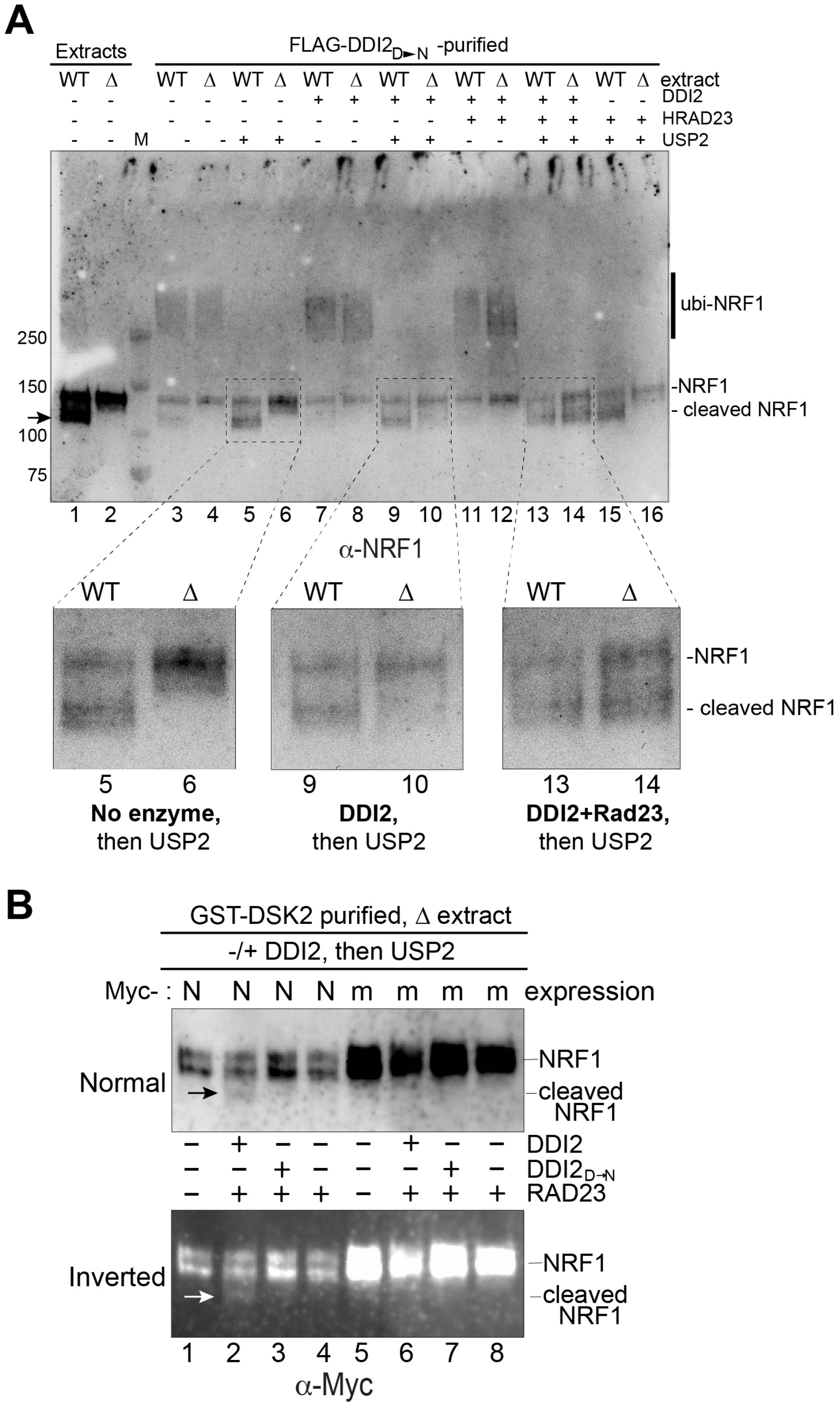

Supplemental Figure S6, related to Figure 4. DDI2 cleaves ubiquitylated NRF1 protein. A. Western blot analysis of NRF1 protein, isolated via FLAG-DDI2$_{D\rightarrow N}$ chromatography, after treatment with DDI2, RAD23, and/or USP2, as indicated. Zoom-in of key lanes 5-6, 9-10, and 13-14 are shown below. Note that, in contrast to GST-DSK2, FLAG-DDI2$_{D\rightarrow N}$ also pulls down small amounts of un-ubiquitylated NRF1 (compare lanes 3-4 with lanes 1-2), suggesting low-affinity, direct binding of DDI2 to NRF1, as well as much higher-affinity binding to the ubiquitylated form. **B.** Western blot analysis of different forms of NRF1 protein (Normal cleavage site, N; mutant cleavage site, m), isolated via GST-DSK2 chromatography, after treatment with DDI2, RAD23, and USP2, as indicated. The mobility of NRF1 and cleaved NRF1 is indicated on the right (see also arrow for cleaved NRF1). Note that no cleavage of mutant NRF1 is detected, in spite of an abundance of substrate (lane 6) relative to normal NRF1 (lane 2). To ease visualization of the weak cleavage observed with these exogenously expressed, myc-tagged proteins, a colour-inverted image of the upper panel is repeated at the bottom.
